# Organic photovoltaics of diketopyrrolopyrrole copolymers with unsymmetric and regiorandom configuration of the side units†

**DOI:** 10.1039/c8ra05903a

**Published:** 2018-08-28

**Authors:** Kenta Aoshima, Marina Ide, Akinori Saeki

**Affiliations:** Department of Applied Chemistry, Graduate School of Engineering, Osaka University 2-1 Yamadaoka Suita Osaka 565-0871 Japan saeki@chem.eng.osaka-u.ac.jp; Precursory Research for Embryonic Science and Technology (PRESTO), Japan Science and Technology Agency (JST) 4-1-8 Honcho Kawaguchi Saitama 332-0012 Japan

## Abstract

Diketopyrrolopyrrole (DPP) is a representative electron acceptor incorporated into narrow-bandgap polymers for organic photovoltaic cells (OPV). Commonly, identical aromatic units are attached to the sides of the DPP unit, forming symmetric DPP polymers. Herein we report the synthesis and characterization of DPP copolymers consisting of unsymmetric configurations of the side aromatics. The unsymmetric DPP copolymer with thienothiophene and benzene side moieties exhibits good solubility owing to the twisted dihedral angle at benzene and regiorandom configuration. A significant shallowing of the highest occupied molecular orbital level is observed in accordance with the electron-donating nature of the side units (benzene, thiophene, and thienothiophene). The overall power conversion efficiencies of the unsymmetric DPPs (2.3–2.4%) are greater than that of the centrosymmetric analogue (0.45%), which is discussed in view of bulk heterojunction morphology, polymer crystallinity, and space-charge-limited current mobilities. This comparative study highlights the effect of unsymmetric design on the molecular stacking and OPV performance of DPP copolymers.

## Abstract 2

Diketopyrrolopyrroles with unsymmetric side aromatics of benzene and (thiophene or thienothiophene) were copolymerized with 2-dimensional benzodithiophene, and their solar cell devices were characterized.

## Graphical abstract

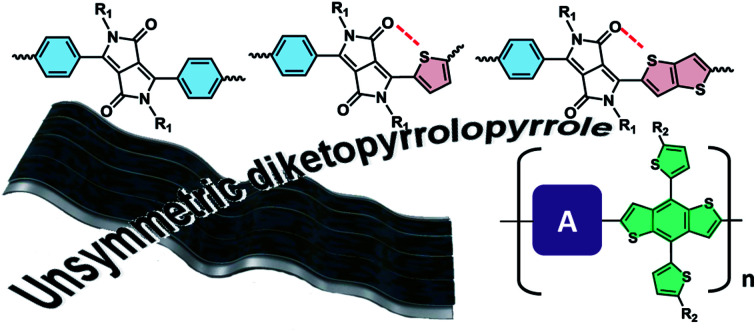

## Introduction

The need to meet a rising global demand for renewable energy sources has led to the exploration of materials for next-generation solar cells. As such, organic photovoltaic cells (OPV) based on a bulk heterojunction (BHJ) framework have been developed,^1–4^ in particular over the last two decades, wherein a bicontinuous network of binary^5–7^ or multiple^8–10^ components consisting of p-type and n-type conjugated materials is central to the photoelectric conversion process. The materials used include polymers and molecules that are mostly composed of covalently-bonded electron-donating and electron-accepting units to manipulate their electrochemical properties such as bandgap (*E*_g_) and intermolecular interactions such as crystallinity^11,12^ and backbone orientation.^13,14^

**Fig. 1:**
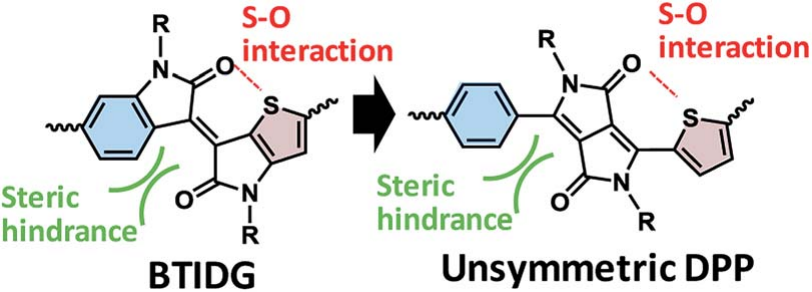
Molecular design of unsymmetric electron-accepting units. (Left) BTDIG, (right) unsymmetric DPP.

Diketopyrrolopyrrole (DPP)^15–23^ and isoindigo (IDG)^24–34^ are relatively strong electron acceptors and have been polymerized into narrow-bandgap polymers for OPV. Based on modification of IDG, Ashraf *et al.*,^35^ Pruissen *et al.*^36^ and Koizumi *et al.*^37^ have reported the synthesis of thienoisoindigo (TIDG) and its copolymers as organic field-effect transistors (OFETs), which is followed by applications of TIDG-based near-infrared (IR)-absorbing copolymers to OPV^38–42^ and OFET.^43–47^ IDG and TIDG have structural similarities to DPPs with symmetric side units of benzene (Ph) or thiophene (Th), respectively. However, TIDG exhibits a stronger electron-accepting tendency than Th-sandwiched DPP because of the direct attachment of Th to the electron-withdrawing ketopyrrole unit, which leads to a greatly narrowed *E*_g_ along with a low open-circuit voltage (*V*_oc_).^38^ Moreover, the most critical issue in the IR-absorbing polymers is that the low short-circuit current density (*J*_sc_) resulting from the energy bandgap law^48^ causes shortening of the exciton lifetime^38^ through increased coupling with the low-energy vibrational mode. To balance the exciton lifetime and optical bandgap, Chen *et al.*^49^ and Ide *et al.*^50,51^ have synthesized a benzothienoisoindigo (BTIDG) unit that replaces one of the Th units of TIDG with Ph (Fig. 1). BTIDG includes an intramolecular attractive S–O interaction, and steric hindrance between the proton of Ph and oxygen of the keto unit, which constitutes a half-distorted π-plane. This unsymmetric molecular design elicited not only a moderate *E*_g_ but also improved film morphology (face-on orientation of polymer backbone and miscible BHJ network), resulting in increased power conversion efficiency (PCE).^50^ Thus, the unsymmetric structure and regiorandom configuration of BTIDG copolymers may contribute to the improved film characteristics associated with their solubilities, in analogy to the unsymmetric DPP polymers having Th and thienothiophene (TT) side units (PCE ∼ 6%).^21^

Herein we report a comparative study on DPP-based copolymers with unsymmetric configurations of Ph and (Th or TT) units. Despite the structural similarity between BTIDG and Ph-DPP-Th (left and right in Fig. 1, respectively), they behave differently. The morphology, polymer orientation, and space-charge-limited current (SCLC) mobilities of DPP-based copolymer-[6,6]-phenyl-C_61_-butyric acid methyl ester (PCBM) blend films are evaluated and discussed, as are their PCEs in an OPV application.

## Results and discussion

**Fig. 2:**
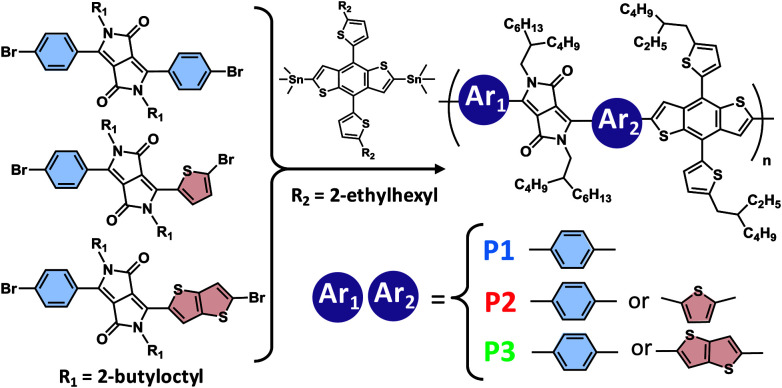
Chemical structures of the unsymmetric, regiorandom DPP-based copolymers. Polymerization was carried out by Stille coupling method using Pd(PPh_3_)_4_ catalyst.

Three DPP copolymers were prepared, including a symmetric Ph-DPP-Ph copolymer (P1) as control, an unsymmetric Ph-DPP-Th copolymer (P2), and an unsymmetric Ph-DPP-TT copolymer (P3), as shown in Fig. 2. Ph-DPP-Ph rather than more planar and common Th-DPP-Th unit^15,17–23,52,53^ was chosen as control in order to examine the change in the backbone planarization from twist (Ph-DPP-Ph) to half-distortion (Ph-DPP-Th (or TT)) (*vide infra*). DPP units were synthesized according to previously described procedures (see Experimental) and polymerized with an electron-donor unit of 2-dimensional benzobisthiophene-appending alkylthiophene side units (BDT-Th) *via* Stille coupling reaction. The resultant unsymmetric DPP polymers are regiorandom, similarly to the pyridylthiadiazole-based molecules and polymers reported by Bazan *et al.*^54,55^ The weight-averaged molecular weights (*M*_w_s) and polydispersity indices (PDIs) of the polymers, as characterized by size exclusion chromatography, were 98.4 kg mol^−1^ (2.7) for P1, 22.2 kg mol^−1^ (2.4) for P2, and 31.6 kg mol^−1^ (2.5) for P3. These polymers showed glass transition at ∼147 °C and the identical profiles during two cycles of differential scanning calorimetry (up to 300 °C), exhibiting no decomposition below this temperature (Fig. S1†). Solubility of P3 was as large as ∼100 mg mL^−1^ even in toluene at room temperature, which is presumably derived from the twisted backbone at the benzene-DPP connection (*vide infra*) and unsymmetric, regiorandom configuration. When comparing solubilities of P1 and P3 in toluene–hexane mixture (1 : 1 vol%), they are well-soluble (∼18 and ∼13 mg mL^−1^, respectively).

**Fig. 3:**
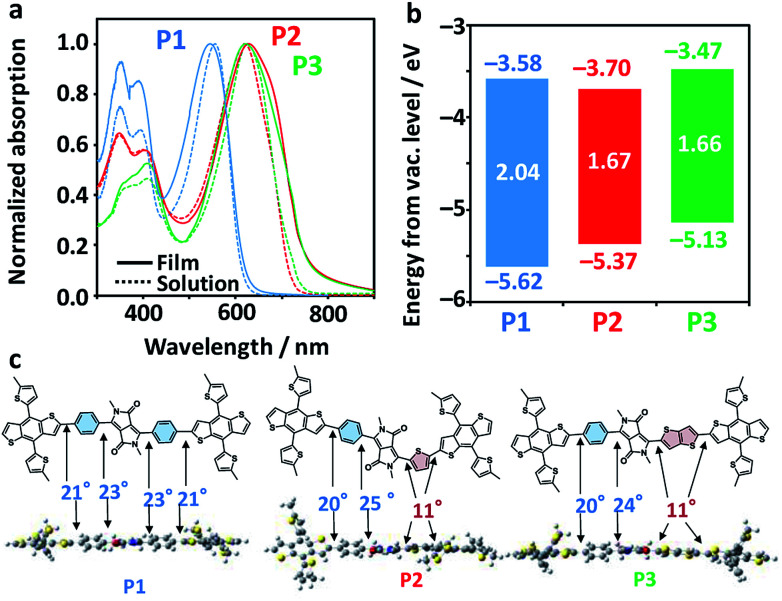
(a) Photoabsorption spectra of P1–P3 in chlorobenzene solutions (dotted lines) and as films (solid lines). (b) Energy diagram of the copolymers. HOMO (the bottom of each bar) and *E*_g_ (the centre of each bar) were evaluated by PYS and the photoabsorption onset in the film, respectively. LUMO (the top of each bar) was calculated by adding *E*_g_ and HOMO. (c) Horizontal view, along with dihedral angles at respective bonds. The donor–acceptor–donor compounds were geometry-optimized using DFT with B3LYP/6-31G*. Alkyl chains were replaced by methyl groups to simplify the calculation.

Fig. 3a shows the photoabsorption spectra of P1–P3 in diluted chlorobenzene solutions and as films. All the polymers exhibited mostly unchanged maxima attributed to an intramolecular charge transfer band in both solution and film phases (545 nm for P1, 629 nm for P2, and 622 nm for P3). Meanwhile, P2 and P3 exhibited accompanying shoulder peaks in the longer-wavelength region, suggestive of their more extensive conjugation systems and higher crystallinities as compared to P1. Fig. 3b displays the electrochemical properties of the polymers, including the highest occupied molecular orbital (HOMO) levels as evaluated using photoelectron yield spectroscopy (PYS) and the *E*_g_s estimated from the photoabsorption spectra of the films (Fig. S2†). The HOMO levels drastically shifted upward, from −5.62 eV for P1, −5.37 eV for P2, and −5.13 eV for P3. The *E*_g_s of P2 and P3 were very similar (1.67 and 1.66 eV, respectively), and are up to 0.4 eV narrower than that of P1 (2.04 eV). The narrowing of the *E*_g_s and shallowing of HOMO levels are rationalized by the order of the electron-donating strengths of Ph, Th, and TT. Density functional theory (DFT) calculations of donor–acceptor–donor units shown in Fig. 3c revealed a contrasting change in the dihedral angle between the BDT-Th donor unit and DPP, where Ph-side angles were almost double (20–25°) those of the Th (or TT)-side angles (11°). The structural planarization of P2 and P3 is likely to partly affect their narrowed *E*_g_s and the appearance of shoulder peaks (improved crystallinity in a film).

**Fig. 4:**
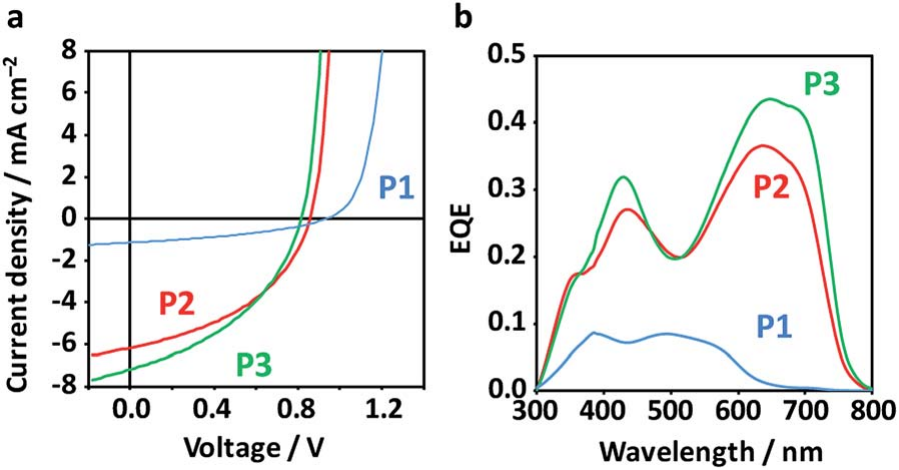
(a) Current density–voltage curves of the best-performing OPV devices under pseudo-sunlight (100 mW cm^−2^). (b) EQE spectra of the corresponding devices.

Inverted-type OPV devices (ITO/ZnO/active layer/MoO_*x*_/Ag) were fabricated from chlorobenzene solutions with 3 vol% 1,8-diiodooctane (DIO) as the solvent additive. We have surveyed the processing conditions including thermal annealing (TA, 120 °C for 10 min) and copolymer : PCBM blend ratios (1 : 1, 1 : 2, and 1 : 3) (Fig. S3 and Table S1†). The highest PCE obtained for P1 was 0.45% (average 0.38 ± 0.04%), while P2 and P3 achieved higher PCEs of 2.30% (average 1.83 ± 0.25%) and 2.40% (average 1.97 ± 0.32%), respectively (Fig. 4a and Table 1). The low PCE of control P1 is linked to the coarse BHJ morphology and low crystallinity (low electron mobility, *vide infra*), while the symmetric copolymer composed of BDT-Th and Th-DPP-Th with the identical alkyl chains have reportedly showed high PCE (1% (ref. 52) and 6.5% (ref. 53)). Despite the low PCE of P1, it exhibited the highest *V*_oc_ (0.99 V) among the polymers (P2: 0.875 V and P3: 0.813 V), which is in accordance with the HOMO levels of the respective polymers. The improvement in PCE for P2 and P3 is mainly caused by the *J*_sc_ (1.25, 6.50, and 7.19 mA cm^−2^ for P1, P2, and P3, respectively), as evident from the large difference in the external quantum efficiency (EQE) spectra (Fig. 4b). The EQE spectra display the same shape as the corresponding photoabsorption spectra of the polymers, and the integrated *J*_sc_ over the EQE spectra are consistent with those under pseudo-sunlight exposure (100 mW cm^−2^). Note that the small *J*_sc_ of P1 is not limited by the narrow *E*_g_, but rather is governed by BHJ morphology (*vide infra*) that simultaneously affects the exciton migration, charge separation, and charge transport. The SCLC hole mobilities (*μ*_h_) of pristine polymers monotonically increased from 2.5 × 10^−6^ cm^2^ V^−1^ s^−1^ for P1 to 1.0 × 10^−5^ cm^2^ V^−1^ s^−1^ for P2, and further to 8.5 × 10^−5^ cm^2^ V^−1^ s^−1^ for P3 (Table S1 and Fig. S4†). In contrast, *μ*_h_s of the optimized blend films were low and mostly comparable (2.8 × 10^−6^ cm^2^ V^−1^ s^−1^ for P1 : PCBM, 2.1 × 10^−6^ cm^2^ V^−1^ s^−1^ for P2 : PCBM, and 2.2 × 10^−6^ cm^2^ V^−1^ s^−1^ for P3 : PCBM), while the SCLC electron mobilities (*μ*_e_) of the blend films increased between P1 (1.4 × 10^−5^ cm^2^ V^−1^ s^−1^) and P2 (1.9 × 10^−4^ cm^2^ V^−1^ s^−1^), and further to P3 (7.9 × 10^−4^ cm^2^ V^−1^ s^−1^) (Table 1 and Fig. S4†). The *μ*_e_ values of P2 : PCBM and P3 : PCBM, which were an order of magnitude larger than that of P1 : PCBM, agrees with the higher crystallinity of the former polymers (*vide infra*). Therefore, *μ*_e_, which is closely related to the purity and percolation network of PCBM domains facilitated by the crystallization process of the polymer in the blends, is likely a dominant factor influencing overall PCE.

**Table 1:** Summary of polymer : PCBM OPV performances^a^ and SCLC mobilities

Polymer (*p* : *n*)	*L*/nm	*J*_sc_ (*J*^EQE^_sc_)^c^/mA cm^−2^	*V*_oc_/V	FF^d^	PCE/%	PCE_ave_^e^/%	*μ*_h_ f/cm^2^ V^−1^ s^−1^	*μ*_e_ f/cm^2^ V^−1^ s^−1^
P1 (1 : 2)^b^	80	1.25 (1.05)	0.992	0.380	0.45	0.38 ± 0.04	2.8 × 10^−6^	1.4 × 10^−5^
P2 (1 : 1)	70	6.50 (6.35)	0.875	0.435	2.30	1.83 ± 0.25	2.1 × 10^−6^	1.9 × 10^−4^
P3 (1 : 1)^b^	80	7.19 (7.48)	0.813	0.411	2.40	1.97 ± 0.32	2.2 × 10^−6^	7.9 × 10^−4^

a Inverted cell (ITO/ZnO/active layer/MoO_*x*_/Ag) under simulated sunlight (100 mW cm^−2^).

b Thermal annealing at 120 °C for 10 min.

c *J* ^EQE^_sc_ is the integrated *J*_sc_ over the EQE spectrum.

d Fill factor.

e Average over at least six devices. The error is a standard deviation.

f SCLC mobility of a hole-only device (*μ*_h_) and an electron-only device (*μ*_e_).

**Fig. 5:**
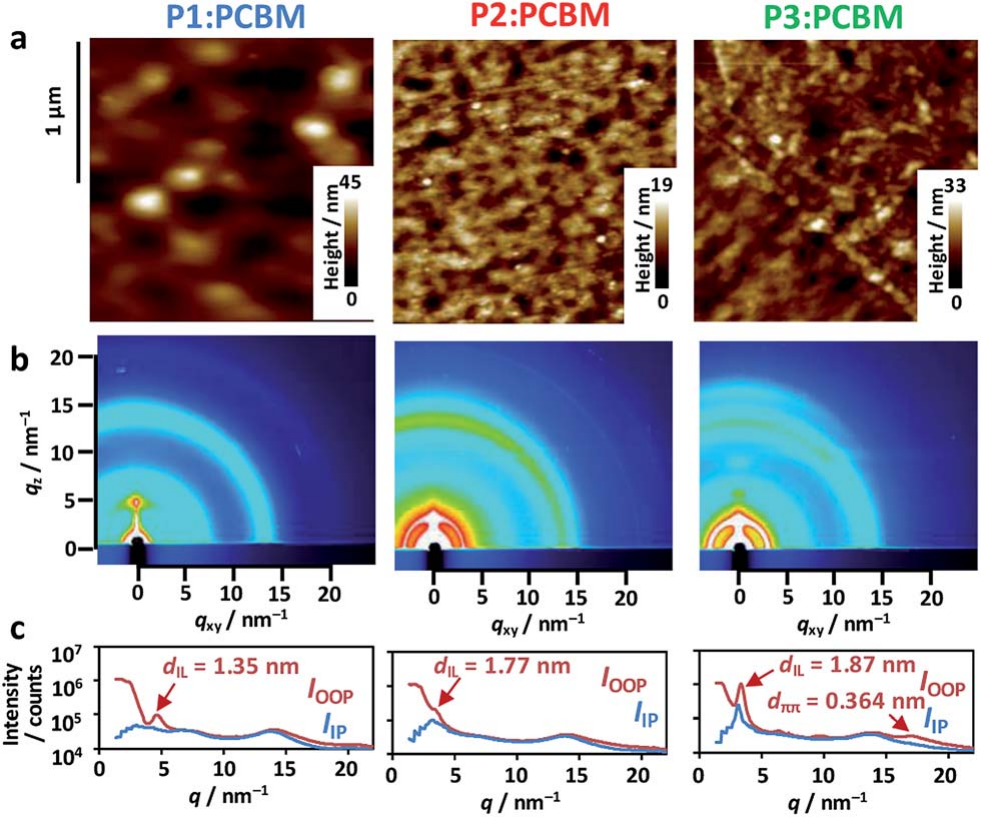
(a) AFM height images of the P1–P3 copolymer : PCBM blend films. (b) 2D-GIXD images of the blend films. (c) Out-of-plane (OOP, red line) and in-plane (IP, blue line) diffraction profiles. The interlamellar (*d*_IL_) and π–π stacking distances (*d*_ππ_) are appended.

Surface morphologies of the blend films were observed by atomic force microscopy (AFM), which revealed large circular grains (∼170 nm diameter) in P1 : PCBM (Fig. 5a). This contrasted the miscible, small grains observed in both P2 : PCBM and P3 : PCBM (∼50 nm diameter). The root mean square (rms) roughness of these height images were mostly comparable: 3.9 nm for P1 : PCBM, 2.0 nm for P2 : PCBM, and 3.1 nm for P3 : PCBM. The 2-dimensional grazing-incidence X-ray diffraction (2D-GIXD) images exhibited weak, less-oriented patterns for all of the blend films (Fig. 5b). The diffraction profile of P3 : PCBM in the out-of-plane (OOP) direction comprises the two peaks attributed to the interlamellar distance (*d*_IL_) of 1.87 nm and π–π stacking distance (*d*_ππ_) of 0.364 nm. The *d*_IL_s of P1 : PCBM and P2 : PCBM were 1.35 and 1.77 nm, respectively, while their π–π stacking peaks in the outer region of the hallow due to the alkyl chains (scattering vector *q* ∼ 14 nm^−1^) were not observed. Regardless of having identical side alkyl chains, the *d*_IL_s of P2 and P3 were larger than that of P1. This is probably due to perturbation of the interdigitation of the side alkyl chains by the winding, regiorandom configuration of the backbone, as seen in the DFT calculations (Fig. 3c). The *d*_IL_ of P1 : PCBM was approximately half the end-to-end distance of the expanded 2-ethylhexyl chains of BDT-Th (∼2.7 nm), indicating that the side chains were well-interdigitated. However, the peak was relatively broad, and the crystallite size calculated using Scherrer's relation^56^ was small (8.4 nm). In contrast, the crystallite sizes of interlamellar and π–π stacking of P3 : PCBM were 13.5 and 3.1 nm, respectively. The former is obviously larger than those of P1 : PCBM (8.4 nm) and P2 : PCBM (11.5 nm). The same *d*_IL_s were obtained in the pristine P1–P3 polymers without PCBM (Fig. S5†). Notably, blending with PCBM increased the interlamellar crystallite sizes of P1–P3 from 4.0 to 8.4 nm, 4.3 to 11.5 nm, and 4.1 to 13.5 nm, respectively (Table S2†). In particular, P3 demonstrated the largest increase, which accompanies the appearance of a π–π stacking peak in the 2D-GIXD profile. Despite the increased crystallinity in the blend films, hole mobilities were decreased and became comparable among the polymers. This may be due to the decreased connectivity of polymer crystallites, suffered from the presence of PCBM domains in BHJ films.

## Conclusions

We synthesized and characterized DPP-BDT-Th copolymers having unsymmetric and regiorandom configurations of the side aromatic rings. The unsymmetric DPP copolymers (P2 and P3) showed greater PCEs (2.3–2.4%) than the symmetric analogue P1 (0.45%), which is mainly attributed to the improved BHJ morphology associated with increased solubility of the unsymmetric polymers. The crystallinities of P2 and P3 in their respective PCBM blends were increased as compared to the corresponding pristine films, leading to enlarged crystallite sizes and relatively good *μ*_e_s. Nonetheless, the insufficient PCEs of P2 and P3 result from the low polymer crystallinities and low *μ*_h_s (10^−6^ cm^2^ V^−1^ s^−1^) in the PCBM blends as compared to the reported symmetric Th-DPP-Th copolymers (PCE = 6–7%).^19,21,53^ The comparative study shows how an unsymmetric molecular design increases the solubility and affects the film morphology and OPV performance of DPP copolymers, broadening the library of conjugated polymers for applications in organic electronics.

## Experimental

### General measurement

Steady-state photoabsorption spectroscopy was performed using a Jasco V-570 UV-vis spectrophotometer. Molecular weights (weight-averaged: *M*_w_) and polydispersity index (PDI) of polymers were determined using the size exclusion chromatography (SEC) (gel permeation chromatography: GPC) method with polystyrene standards. SEC-GPC analysis was performed with chloroform as an eluent at a flow rate of 1 cm^3^ min^−1^ at 40 °C, on a SHIMADZU LC-20AT, CBM-20A, CTO-20A chromatography instrument connected to a SHIMADZU SPD-M20A UV-vis detector. Photoelectron yield spectroscopy (PYS) of the polymer films on indium-tin-oxide (ITO) glass was performed on a Bunko Keiki BIP-KV2016K instrument. 2D-GIXD experiments were conducted at the SPring-8 on the beam line BL46XU using 12.39 keV (*λ* = 1 Å) X-ray. The GIXD patterns were recorded with a 2-D image detector (Pilatus 300K). Atomic force microscopy (AFM) was carried out on a Bruker Innova AFM microscope. Differential scanning calorimetry (DSC) was performed using a Netzsch model DSC204F1 Phoenix under N_2_ at 10 °C min^−1^ (sample weight = 2.1–2.9 mg). Film thicknesses were measured using a Bruker Dektak XT surface profiler. Solubility of the polymers were measured by dissolving weighted copolymers in chlorobenzene at 80 °C, cooling down to room temperature, filtrating (0.20 μm) the solution, weighting the remained copolymer in a filter, and measuring the volume of the solution.

### Synthesis of polymers

**Fig. 6:**
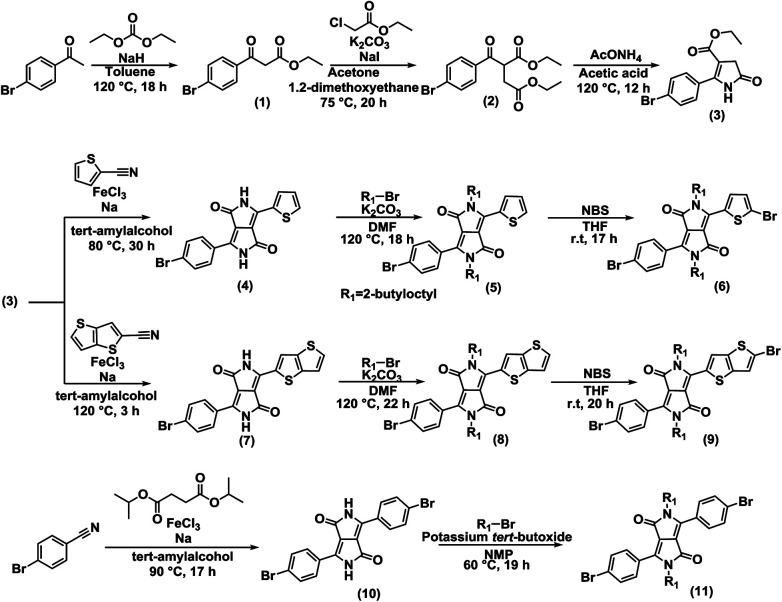
Synthesis of symmetric and unsymmetric DPP monomers. The enlarged figure and details of the synthesis are provided in ESI.†

Symmetric and unsymmetric DPP monomers were synthesized according to the references (Fig. 6).^21,57–61^ The details of the synthesis are provided in ESI.† The polymers (P1, P2, and P3) were synthesized *via* Stille coupling using (PPh_3_)_4_Pd catalyst from the relevant Br-DPP-Br monomer and (Me_3_Sn)-(BDT-2Th)-(SnMe_3_) monomer (Fig. 2). The yields were 36% for P1, 18% for P2, and 75% for P3. The ^1^H NMR spectra of monomers (Fig. S6–S8) and polymers (Fig. S9) are provided in ESI.†

### Organic photovoltaic cell (OPV)

A ZnO layer was fabricated onto a cleaned ITO layer by spin-coating with a ZnO precursor solution (0.1 g mL^−1^ zinc acetate dihydrate and 0.028 g mL^−1^ ethanolamine in 2-methoxyethanol). The substrate was annealed on a hot plate at 200 °C for 30 min. An active layer was cast on top of the ZnO layer in a nitrogen glove box by spin-coating. An anode consisting of 10 nm MoO_*x*_ and 100 nm Ag layers was sequentially deposited on top of the active layers, through a shadow mask, by thermal evaporation in a vacuum chamber. The resulting device configuration was an ITO (120–160 nm)/ZnO (30 nm)/active layer/MoO_*x*_ (10 nm)/Ag (100 nm) with an active area of 7.1 mm^2^. Current density–voltage curves were measured using a source-measure unit (ADCMT Corp., 6241A) under AM 1.5 G solar illumination at 100 mW cm^−2^ (1 sun, monitored by a calibrated standard cell, Bunko Keiki SM-250KD) from a 300 W solar simulator (SAN-EI Corp., XES-301S). The EQE spectra were measured by a Bunko Keiki model BS-520BK equipped with a Keithley model 2401 source meter. The monochromated light power was calibrated by a silicon photovoltaic cell, Bunko Keiki model S1337-1010BQ.

### Space-charge-limited current (SCLC)

The SCLC device structures consisted of an ITO/PEDOT:PSS/active layer (100–200 nm)/Au for the hole, and Al/active layer (∼200 nm)/LiF/Al for the electron. The other procedures are similar to those of the OPV device. The mobility was determined by fitting a current density–voltage curve into the Mott–Gurney law, *J* = 9*ε*_0_*ε*_r_*μV*^2^(8*L*^3^)^−1^, where *ε*_0_ is the permittivity of free space, *ε*_r_ is the dielectric constant of the material, *μ* is the mobility, *V* is the voltage drop across the device, and *L* is the thickness of the active layer.

## Conflicts of interest

There are no conflicts to declare.

## Supplementary Material

RA-008-C8RA05903A-s001
