# Supplementary material for: Organic photovoltaics of diketopyrrolopyrrole copolymers with unsymmetric and regiorandom configuration of the side units
Source: RSC Adv. 2018 Aug 28;8(53):30201–6. doi: 10.1039/c8ra05903a (PMC9085421; doi:10.1039/c8ra05903a)
Supplement: RA-008-C8RA05903A-s001 [file RA-008-C8RA05903A-s001.pdf]

Electronic Supplementary Material (ESI) for RSC Advances.
This journal is © The Royal Society of Chemistry 2018

Electronic Supplementary Information (ESI)

Organic Photovoltaics of Diketopyrrolopyrrole Copolymers with Unsymmetric and Regiorandom Configuration of the Side Units

Kenta Aoshima,[a] Marina Ide,[a] and Akinori Saeki[*, a, b]*

[a]Department of Applied Chemistry, Graduate School of Engineering, Osaka University, 2-1 Yamadaoka, Suita, Osaka 565-0871, Japan.

[b]Precursory Research for Embryonic Science and Technology (PRESTO), Japan Science and Technology Agency, 4-1-8 Honcho, Kawaguchi, Saitama 332-0012, Japan.

AUTHOR INFORMATION

Corresponding Author

*saeki@chem.eng.osaka-u.ac.jp (A.S.)

This supporting information presents the following contents.

Experimental

Supplementary References

Supplementary Table S1–S3

Supplementary Figs. S1–S9

Experimental

Synthesis of monomer

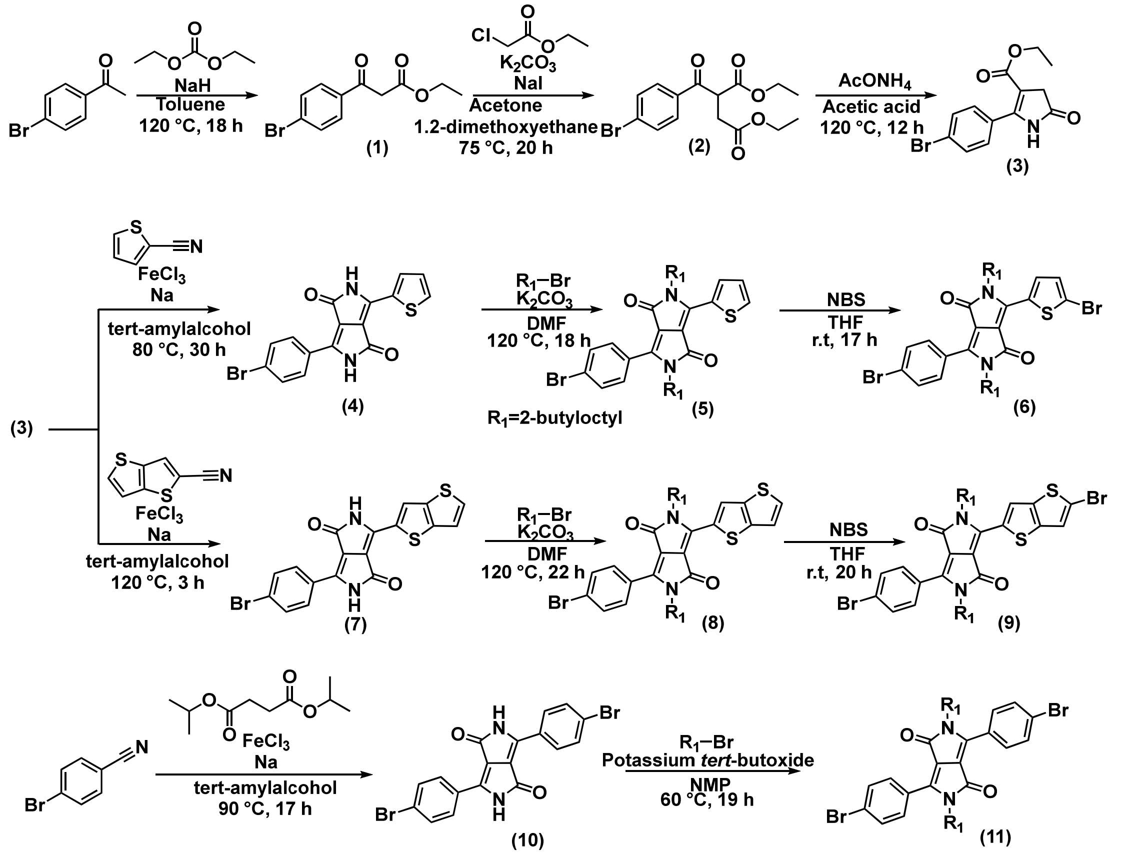

Scheme 1. Synthesis of monomer.

Ethyl 3-(4'-bromophenyl)-3-oxopropionate (1).[1] Sodium hydride (60% oil dispersion, 1.39 g, 3.47 × 10^{-2} mol), 4'-bromoacetophenone (2.66 g, 1.34 × 10^{-2} mol) and Toluene (60 mL) were added to a 100 mL dried flask at room temperature under nitrogen atmosphere. Diethyl carbonate (9.53 mL, 7.88 × 10^{-2} mol) in toluene (20 mL) was added dropwise to the flask with stirring. Then the reaction mixture was refluxed for 18 h. After cooling to room temperature, the mixture was then poured over ice (100 mL) and conc. HCl (4 mL). The aqueous layer was washed with ethyl acetate. The combined organic layers were washed with cold H_2O and brine. The solution was dried over anhydrous $MgSO_4$, filtered, and the solvent was removed under reduced pressure. The crude oil was purified by silica-gel column chromatography using Hexane/Ethyl acetate (9/1 v/v) as the eluent to obtain brown oil (2.55 g, 9.42 × 10^{-3} mol, 71 % yield).

^{1}H NMR (400 MHz, $CDCl_3$), δ 1.24 (t, J = 7.2 Hz, 3H), 3.94 (s, 2H), 4.21 (q, J = 7.2 Hz, 2H), 7.63 (d, J = 8.4 Hz, 2H), 7.81 (d, J = 8.8 Hz, 2H).

Butanedioic acid, 2-(4-bromobenzoyl)-, 1,4-diethyl ester (2).[2] **(1)** (3.66 g, 1.35 × 10^{-2} mol), potassium carbonate (1.86 g, 1.34 × 10^{-2} mol), sodium iodide (0.255 g, 1.71 × 10^{-3} mol), acetone (6.75 mL) and 1,2-dimethoxyethane (4.82 mL) were added to a 50 mL dried flask at room temperature under nitrogen. Ethyl chloroacetate (1.33 mL, 1.35 × 10^{-2} mol) was added dropwise to the flask with stirring. Then the reaction mixture was stirred for 20 h at 75°C. After cooling to room temperature, the mixture was quenched by H_2O and extracted with Chloroform. The organic layer was washed with brine and dried over anhydrous $MgSO_4$, filtered, and the solvent was removed under reduced pressure. The residue was purified by silica-gel column chromatography using Hexane/Ethyl acetate (8/1 v/v) as the eluent to obtain yellow oil (2.73 g, 7.66 × 10^{-3} mol, 57 % yield).

5-oxo-2-(4-bromophenyl)-4.5-dihydropyrrole-3-carboxylic acid ethyl ester (3).[2] **(2)** (2.81 g, 7.87 × 10^{-3} mol), ammonium acetate (6.06 g, 7.87 × 10^{-2} mol), and acetic acid (15.7 mL) were added to 50 mL dried flask at room temperature under nitrogen. Then the reaction mixture was stirred for 6 h at 120°C. After cooling to room temperature, the mixture was poured into H_2O. The precipitate was filtered, and washed with water and methanol. The residue was dried under reduced pressure to afford grayish blue solid (1.16 g, 3.74 × 10^{-3} mol, 48 % yield).
^{1}H NMR (400 MHz, DMSO-d$_6$), δ 1.09 (t, J = 7.2 Hz, 3H), 3.37 (s, 2H), 4.00 (q, J = 7.2 Hz, 2H), 7.52 (d, J = 8.8 Hz, 2H), 7.66 (d, J = 8.8 Hz, 2H), 10.68 (s, 1H)

3-(4-bromophenyl)-6-(thiophen-2-yl)-2,5-dihydropyrrolo[3,4-c]pyrrole-1,4-dione (4).[2] Sodium (0.650 g, 2.35 × 10^{-2} mol), $FeCl_3$ (7.5 mg, 4.65 × 10^{-5} mol) and *tert*-amylalcohol (22.5 mL) were added to 50 mL dried flask at room temperature under nitrogen. Then the reaction mixture was stirred at 100°C until sodium dissolved. **(3)** (2.35 g, 7.50 × 10^{-3} mol) and 2-cyanothiophene (1.00 g, 9.16 × 10^{-3} mol) were added and the mixture was heated to 80°C and stirred at the temperature for 6 h. Then, acetic acid (5 mL) and methanol (40 mL) were added and the mixture was cooled to room temperature, and then poured into methanol (100 mL). The precipitate was filtered and washed H_2O and Methanol. The residue was dried under reduced pressure to afford red solid and was used for next reaction without further purification (1.80 g, 4.81 × 10^{-3} mol, 53 % yield).
^{1}H NMR (400 MHz, DMSO-d$_6$), δ 7.30-8.40 (m, 7H), 11.3 (s, 1H), 11.4 (s, 1H).

2,5-bis(2-butyloctyl)-3-(4-bromophenyl)-6-(thiophen-2-yl)-2,5-dihydropyrrolo[3,4-c]pyrrole-1,4-dione (5).[3] **(4)** (1.00 g, 2.68 × 10^{-3} mol), 1-bromo-2-butyloctane (1.67 g, 6.70 × 10^{-3} mol), potassium carbonate (1.11 g, 8.04 × 10^{-3} mol) and 18-crown-6 (22 mg) were placed in 100 mL dried flask at room temperature under nitrogen. *N,N*-dimethylformamide (47.9 ml) was added to the flask and then the reaction mixture was stirred for 19 h at 120°C. The reaction mixture was cooled to room temperature, quenched by H_2O and extracted with hexane/ethyl acetate (4/1 v/v). The organic layer was dried over anhydrous $MgSO_4$, filtered, and the solvent was removed under reduced pressure. The residue was purified by silica-gel column chromatography using hexane/ethyl acetate (10/1 v/v) as the eluent to obtain bright red oil (0.684 g, 9.64 × 10^{-4} mol, 36 % yield).
^{1}H NMR (400 MHz, acetone-d$_6$), δ 0.76-0.89 (m, 12H), 1.05-1.36 (m, 36H), 1.52 (m, 1H), 1.89 (m, 1H), 3.88 (d, J = 7.2 Hz, 2H), 3.99 (d, J = 8.0 Hz, 2H), 7.40 (m, 1H), 7.78-7.89 (m, 4H), 8.02 (m, 1H), 8.98 (m, 1H).

2,5-bis(2-butyloctyl)-3-(4-bromophenyl)-6-(5-bromothiophen-2-yl)-2,5-dihydropyrrolo[3,4-c]pyrrole-1,4-dione (6).[2] **(5)** (0.684 g, 9.64 × 10^{-4} mol) and *N*-Bromosuccinimide (0.171 g, 9.64 × 10^{-4} mol) were placed in 50 mL dried flask at room temperature under nitrogen. Tetrahydrofuran (16.0 mL) was added to the flask and the reaction mixture was stirred for 17 h at room temperature, and then quenched by H_2O and extracted with chloroform. The organic layer was dried over anhydrous $MgSO_4$, filtered, and the solvent was removed under reduced pressure. The residue was purified by silica-gel column chromatography using hexane/ethyl acetate (47/3 v/v) as the eluent to obtain bright red oil (0.366 g, 9.64 × 10^{-4} mol, 48 % yield).
^{1}H NMR (400 MHz, acetone-d$_6$), δ 0.76–0.89 (m, 12H), 1.05–1.36 (m, 36H), 1.50 (m, 1H), 1.87 (m, 1H), 3.87 (d, J = 7.2 Hz, 2H), 3.94 (d, J = 8.0 Hz, 2H), 7.47 (m, 1H), 7.76–7.98 (m, 4H), 8.67 (d, J = 4.0 Hz, 1H). See Fig. S7 for the spectrum.

3-(4-bromophenyl)-6-(thieno[3,2-b]thiophen-2-yl)-2,5-dihydropyrrolo[3,4-c]pyrrole-1,4-dione (7).[2,3] Sodium (0.469 g, 2.16 × 10^{-2} mol), $FeCl_3$ (7.3 mg, 4.50 × 10^{-5} mol) and *tert*-amylalcohol (28.5 mL) were added to 50 mL dried flask at room temperature under nitrogen. Then the reaction mixture was stirred for 2 h at 95°C until sodium dissolved. **(3)** (2.00 g, 6.45 × 10^{-3} mol) and 2-cyanothieno[3,2-*b*]thiophene (1.19 g, 7.20 × 10^{-3} mol) were added and the mixture was heated to 120°C and stirred at the temperature for 3 h. Then, acetic acid (10 mL) and methanol (40 mL) were added and the mixture was stirred at 120°C for 30 min, cooled to room temperature, and then poured into methanol (100 mL). The precipitate was filtered and washed H_2O and methanol. The residue was dried under reduced pressure to afford dark purple solid and was used for next reaction without further purification (1.72 g, 4.00 × 10^{-3} mol, 56 % yield).

1-bromo-2-butyloctane.[4] 2-butyloctanol (3.49 g, 1.88 × 10^{-2} mol), triphenylphosphine (9.86 g, 3.96 × 10^{-2} mol) and dichloromethane (12.8 mL) was added to 50 mL dried flask at room temperature under nitrogen atmosphere. The reaction mixture was cooled to 0°C and *N*-bromosuccinimide (5.02 g, 2.82 × 10^{-2} mol) was added portionwise. Then the reaction mixture was stirred for 28 h at room temperature, quenched by H_2O and extracted with Hexane. The organic layer was dried over anhydrous $MgSO_4$, filtered, and the solvent was removed under reduced pressure. The residue was purified by silica-gel column chromatography using Hexane as the eluent to obtain colorless oil (2.75 g, 1.10 × 10^{-2} mol, 59 % yield).
^{1}H NMR (400 MHz, $CDCl_3$), δ 0.84–0.92 (m, 6H), 1.22–1.38 (m, 16H), 1.59 (m, 1H), 3.45 (d, *J* = 4.4 Hz, 2H)

2,5-bis(2-butyloctyl)-3-(4-bromophenyl)-6-(thieno[3,2-b]thiophene-2-yl)-2,5-dihydropyrrolo[3,4-c]pyrrole-1,4-dione (8).[3] **(7)** (0.943 g, 2.20 × 10^{-3} mol), 1-bromo-2-butyloctane (1.37 g, 5.49 × 10^{-3} mol), potassium carbonate (0.91 g, 6.59 × 10^{-3} mol) and 18-crown-6 (10 mg) were placed in 100 mL dried flask at room temperature under nitrogen. *N*,*N*-dimethylformamide (39.3 ml) was added to the flask and then the reaction mixture was stirred for 14 h at 120°C. The reaction mixture was cooled to room temperature, quenched by H_2O and extracted with hexane/ethyl acetate (4/1 v/v). The organic layer was dried over anhydrous $MgSO_4$, filtered, and the solvent was removed under reduced pressure. The residue was purified by silica-gel column chromatography using hexane/ethyl acetate (10/1 v/v) as the eluent to obtain red purple oil (0.379 g, 4.95 × 10^{-4} mol, 22 % yield).
^{1}H NMR (400 MHz, acetone-d_6), δ 0.78-0.88 (m, 12H), 1.07–1.38 (m, 36H), 1.51 (m, 1H), 1.89 (m, 1H), 3.90 (d, *J* = 7.6 Hz, 2H), 4.02 (d, *J* = 7.6 Hz, 2H), 7.58–7.61 (m, 1H), 7.78–7.90 (m, 4H), 7.93–7.96 (m, 1H), 9.30 (s, 1H).

2,5-bis(2-butyloctyl)-3-(4-bromophenyl)-6-(5-bromothieno[3,2-b]thiophene-2-yl)-2,5-dihydropyrrolo[3,4-c]pyrrole-1,4-dione (9).[3] **(8)** (0.379 g, 4.95 × 10^{-4} mol) and *N*-Bromosuccinimide (0.088 g, 4.94 × 10^{-4} mol) were placed in 50 mL dried flask at room temperature under nitrogen. Tetrahydrofuran (8.25 mL) was added to the flask and the reaction mixture was stirred for 20 h at room temperature, and then quenched by H_2O and extracted with chloroform. The organic layer was dried over anhydrous $MgSO_4$, filtered, and the solvent was removed under reduced pressure. The residue was purified by silica-gel column chromatography using hexane/ethyl acetate (10/1 v/v) as the eluent to obtain red purple oil (0.189 g, 2.24 × 10^{-4} mol, 45 % yield).
^{1}H NMR (400 MHz, acetone-d_6), δ 0.78–0.91 (m, 12H), 1.07–1.37 (m, 36H), 1.51 (m, 1H), 1.89 (m, 1H), 3.88 (d, *J* = 7.6 Hz, 2H), 3.98 (d, *J* = 8.0 Hz, 2H), 7.73 (s, 1H), 7.75–7.87 (m, 4H), 9.12 (s, 1H). See Fig. S8 for the spectrum.

3,6-bis(4-bromophenyl)pyrrolo[3,4-c]pyrrole-1,4-dione (10).[5] Sodium (0.737 g, 3.20 × 10^{-2} mol) and $FeCl_3$ (13.0 mg, 8.00 × 10^{-5} mol) were placed in 50 mL dried flask at room temperature under nitrogen. 2-methyl-2-butanol (23.4 mL) was added to the flask, and the reaction mixture was stirred at 90°C for 15 min. After Sodium was completely dissolved, the reaction mixture was cooled to 50°C. Then 4-bromobenzonitrile (2.91 g, 1.60 × 10^{-2} mol) was added at the temperature. Dimethyl succinate (1.62 g, 8.01 × 10^{-3} mol) in 2-methyl-2-butanol (5 mL) was added dropwise, and the reaction mixture was stirred at 90°C for 16 h. The reaction mixture was cooled to room temperature and acetic acid (8 mL) and methanol (8 mL) were added to the flask, then the mixture was filtered. The filtered solid was washed with H_2O and methanol and dried under reduced pressure to obtain dark red solid and was used without further purification (2.38 g, 5.33 × 10^{-3} mol, 66 % yield).

3,6-bis(4-bromophenyl)-2,5-bis(2-butyloctyl)pyrrolo[3,4-c]pyrrole-1,4-dione (11).[6] **(10)** (0.545 g, 1.12 × 10^{-3} mol), 1-bromo-2-butyloctane (1.41 g, 5.65 × 10^{-3} mol), potassium *tert*-butoxide (0.313 g, 2.79×10^{-3} mol) were placed in 20 mL dried flask at room temperature under nitrogen. *N*-methylpyrrolidone (8.6 mL) was added to the flask and then the reaction mixture was stirred for 20 h at 60°C. The reaction mixture was cooled to room temperature, quenched by H_2O and extracted with toluene. The organic layer was dried over anhydrous $MgSO_4$, filtered, and the solvent was removed under reduced pressure. The residue was purified by silica-gel column chromatography using hexane/dichloromethane (3/2 v/v) as the eluent and recrystallization (methanol and chloroform) to obtain orange crystal (0.108 g, 1.38 × 10^{-4} mol, 12 % yield).
^{1}H NMR (400 MHz, $CDCl_3$), δ 0.81–1.34 (m, 48H), 1.51 (m, 2H), 3.71 (d, *J* = 7.6 Hz, 4H), 7.65 (m, 8H). See Fig. S8 for the spectrum.

Synthesis of polymers

P1. To a degassed solution of the monomer **(11)** (80.0 mg, 1.02 × 10^{-4} mol), 4,8-bis[5-(2-ethylhexyl)thiophen-2-yl]-2,6-bis(trimethylstannyl)benzo[1,2-*b*:4,5-*b'*]dithiophene (92.5 mg, 1.02 × 10^{-4} mol) of toluene (1.86 mL) and DMF (0.186 mL) in 30 mL flask, tetrakis(Triphenylphosphine)palladium(0) (5.91 mg, 5.11 × 10^{-6} mol) were added. The mixture was stirred at 120°C for 17 h, after which bromobenzene (0.1 mL) and trimethyltin benzene (0.1 mL) were added to end-cap the reaction point. The reaction mixture was precipitated in methanol and filtered to obtain polymer. The polymer was extracted with methanol, acetone, hexane and then dissolved in chloroform at boiling point, which was then precipitated in acetone to obtain red polymer (61.4 mg, 36% yield). M_w = 98.4 kg mol^{-1}, PDI = 2.7. Elementary analysis; C: 75.36% (calc:76.00), H: 8.35% (calc:8.39%), N: 2.22 (calc:2.33%), S: 10.49% (calc:10.67%).

P2. To a degassed solution of the monomer **(6)** (170.0 mg, 2.16 × 10^{-4} mol), 4,8-bis[5-(2-ethylhexyl)thiophen-2-yl]-2,6-bis(trimethylstannyl)benzo[1,2-*b*:4,5-*b'*]dithiophene (194.0 mg, 2.14 × 10^{-4} mol) of toluene (3.93 mL) and DMF (0.393 mL) in 20 mL Schlenk flask, tetrakis(Triphenylphosphine)palladium(0) (12.54 mg, 1.08 × 10^{-5} mol) were added. The mixture was stirred at 115°C for 18 h, after which bromobenzene (0.1 mL) and trimethyltin benzene (0.1 mL) were added to end-cap the reaction point. The reaction mixture was precipitated in methanol and filtered to obtain polymer. The polymer was extracted with methanol, acetone, hexane and then dissolved in chloroform at boiling point, which was then precipitated in acetone to obtain blue powder polymer (66.8 mg, 18% yield). M_w = 22.2 kg mol^{-1}, PDI = 2.4. Elementary analysis; C: 69.79% (calc:73.60), H: 7.64% (calc:8.18%), N: 1.89 (calc:2.32%), S: 13.57% (calc:13.3%).

P3. To a degassed solution of the monomer **(9)** (189.1 mg, 2.24 × 10^{-4} mol), 4,8-bis[5-(2-ethylhexyl)thiophen-2-yl]-2,6-bis(trimethylstannyl)benzo[1,2-*b*:4,5-*b'*]dithiophene (202.6 mg, 2.24 ×10^{-4} mol) of xylene (4.07 mL) and DMF (0.407 mL) in 20 mL Schlenk flask, tetrakis(Triphenylphosphine)palladium(0) (12.9 mg, 1.12 × 10^{-5} mol) were added. The mixture was stirred at 115°C for 18 h, after which bromobenzene (0.1 mL) and trimethyltin benzene (0.1 mL) were added to end-cap the reaction point. The reaction mixture was precipitated in methanol and filtered to obtain polymer. The polymer was extracted with methanol, acetone, hexane and then dissolved in chloroform at boiling point, which was then precipitated in acetone to obtain blue powder like solid (294 mg, 75% yield). M_w = 31.6 kg mol^{-1}, PDI = 2.5. Elementary analysis; C: 71.80% (calc:72.20), H: 7.53% (calc:7.82%), N: 1.88 (calc:2.22%), S: 14.26% (calc:15.2%).

Supplementary References

1. M A. Evans, D. C. Smith, J. M. Holub, A. Argenti, M. Hoff, G. A. Dalglish, D. L.Wilson, B. M. Taylor, J. D. Berkowitz, B. S. Burnham, K. Krumpe, J. T. Gupton, T. C. Scarlett, R. W. Durham, Jr and I. H. Hall, *Arch. Pharm. Pharm. Med. Chem.* 2003, **336**, 181.
2. Y. Xu, Y. Jin, W. Lina, J. Peng, H. Jiang and D. Cao, *Synth. Met.*, 2010, **160**, 2135–2142.
3. Y. Ji, C. Xiao, Q. Wang, J. Zhang, C. Li, Y. Wu, Z. Wei, X. Zhan, W. Hu, Z. Wang, R. A. J. Janssen and W. Li, *Adv. Mater.,* 2016, **28**, 943.
4. X. Guo, R. P. Ortiz, Y. Zheng, M.-G. Kim, S. Zhang, Y. Hu, G. Lu, A. Facchetti and T. J. Marks, *J. Am. Chem. Soc.*, 2011, **133**, 13685–13697.
5. K. Chung, M. S. Kwon, B. M. Leung, A. G. Wong-Foy, M. S. Kim, J. Kim, S. Takayama, J. Gierschner, A. J. Matzger and J. Kim, *ACS Cent. Sci.*, 2015, **1**, 94–102.
6. M. Sasikumar, D. Bharath, G. S. Kumar, N. R. Chereddya, S. Chithiravel, K. Krishnamoorthy, B. Shanigaram, K. Bhanuprakash, V. J. Rao, *Synth. Met.,* 2016, **220**, 236–246.

Supplementary Tables

Table S1. Summary of the polymer:PCBM OPV performances.[a]

Polymer	p:n	TA[b]	L / nm	J_{sc} / mA cm^{-2}	V_{oc} / V	FF	PCE /%	PCE_{ave} /%[c]
P1	1 : 1	–	130	0.90	0.932	0.389	0.20	0.13 ± 0.05
	1 : 1	120°C	130	0.86	0.781	0.338	0.13	0.12 ± 0.02
	1 : 2	–	140	0.90	0.748	0.372	0.23	0.20 ± 0.03
	1 : 2	120°C	150	0.89	1.044	0.514	0.37	0.29 ± 0.06
	1 : 2	120°C	80	1.25	0.992	0.380	0.45	0.38± 0.04
	1 : 3	–	170	0.70	0.619	0.374	0.16	0.15 ± 0.01
	1 : 3	120°C	170	0.72	0.991	0.421	0.30	0.25 ± 0.05
P2	1 : 1	–	70	6.50	0.875	0.435	2.30	1.83 ± 0.25
	1 : 1	120°C	70	5.00	0.702	0.363	0.89	0.53 ± 0.22
	1 : 2	–	80	3.63	0.874	0.433	1.31	1.14 ± 0.14
	1 : 2	120°C	80	2.44	0.875	0.407	0.87	0.70 ± 0.24
	1 : 3	–	140	3.55	0.841	0.436	1.28	1.11 ± 0.14
	1 : 3	120°C	130	3.43	0.763	0.442	1.11	0.98 ± 0.09
P3	1 : 1	–	60	6.77	0.799	0.367	1.95	1.55 ± 0.23
	1 : 1	120°C	80	7.19	0.813	0.411	2.40	1.97 ± 0.32
	1 : 2	–	130	6.86	0.711	0.432	2.07	1.96 ± 0.12
	1 : 2	120°C	100	7.38	0.793	0.437	2.29	2.12 ± 0.21
	1 : 3	–	140	6.03	0.830	0.400	1.94	1.81 ± 0.12
	1 : 3	120°C	130	6.32	0.757	0.439	2.04	1.92 ± 0.13

[a] Inverted cell (ITO/ZnO/Active layer/MoO_x/Ag) under 1 sun (100 mW cm^{-2}). [b] Thermal annealing (TA) at 120°C for 10 min. [c] Averaged PCE over at least six devices. The best PCE for each polymer is highlighted by color.

Table S2. Analysis of the out-of-plane profiles of 2D-GIXD.

Polymer	Blend [a]	d_{IL} / nm	Crystallite size / nm	$d_{\pi\pi}$ / nm	Crystallite size / nm
P1	Pristine	1.26	4.0	–	–
	Blend	1.35	8.4	–	–
P2	Pristine	1.60	4.3	–	–
	Blend	1.77	11.5	–	–
P3	Pristine	1.87	4.1	–	–
	Blend	1.87	13.5	0.364	3.1

[a] Blend means OPV-optimal blend ratio of PCBM (film thickness: 70–80 nm).

Table S3. SCLC mobilities of the hole-only devices of pristine polymers.[a]

	P1	P2	P3
μ_h / cm^{2}V^{-1}s^{-1}	2.5×10^{-6}	1.0×10^{-5}	8.5×10^{-5}

[a] ITO/PEDOT/polymer/Au.

Supplementary Figures.

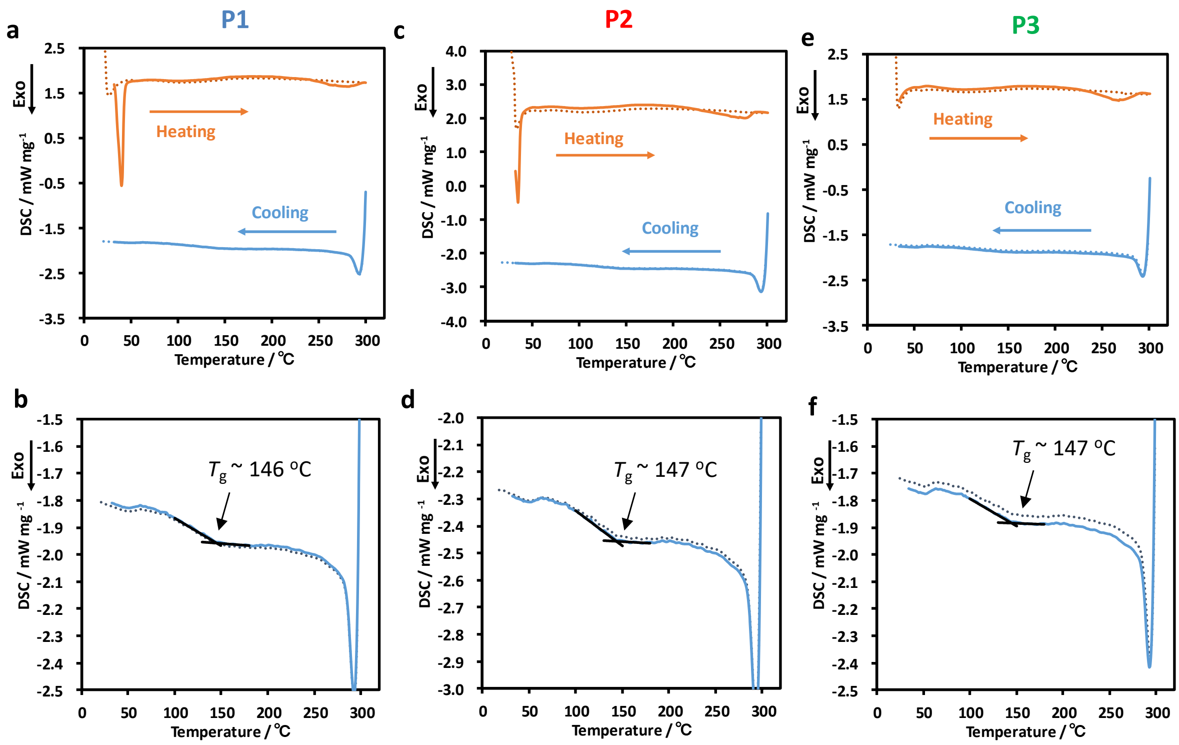

Fig. S1. DSC profiles of (a)(b) P1, (c)(d) P2, and (e)(f) P3. The orange solid and dotted lines represent heating from 20 °C to 300 °C, while the blue solid and dotted lines correspond to cooling from 300 °C to 20 °C. The coloured solid and dotted line represent the first heating/cooling and the second heating/cooling, respectively. Rate: 10 °C min^{-1}. The sample weights: P1 = 2.890 mg, P2 = 2.119 mg, and P3 = 2.796 mg.

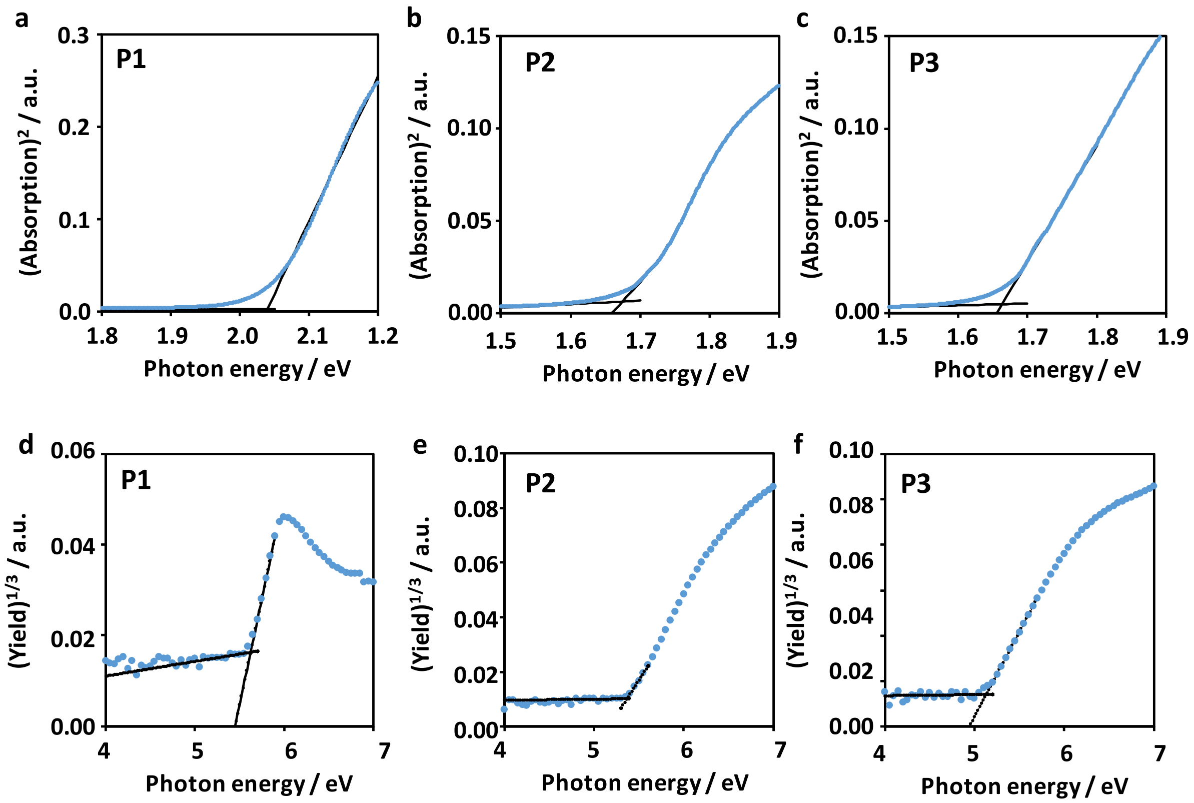

Fig. S2. (a)-(c) Photoabsorption spectra for the evaluation of E_g. (d)-(f) PYS spectra for the evaluation of HOMO level. These spectra were measured in the films. The black lines are extrapolated lines.

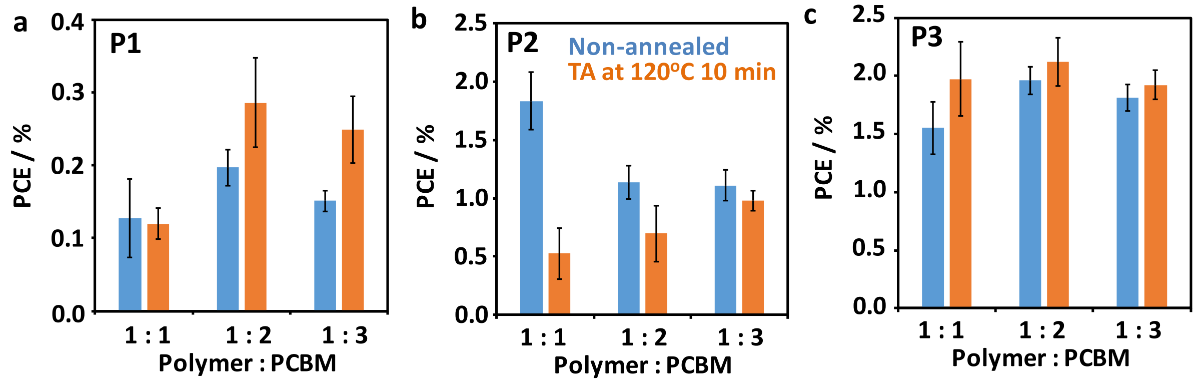

Fig. S3. Bar plots of the average OPV performances of (a) P1:PCBM, (b) P2:PCBM, and (c) P3:PCBM. The data are the same with Table S1. The blue and orange bars in (a)-(c) represent non-thermal annealing and thermal annealing (TA) at 120°C for 10 min, respectively. The black bars are error bars derived from the statistics.

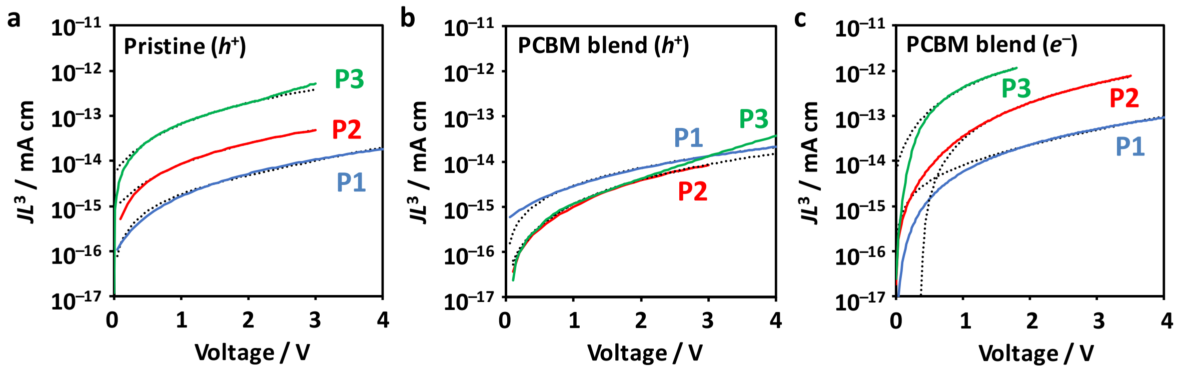

Fig. S4. SCLC curves of (a) hole-only devices of pristine polymer, (b) hole-only devices of polymer:PCBM films, and (c) electron-only devices of polymer:PCBM films. The black dotted lines are fitting curves based on Mott-Gurney law.

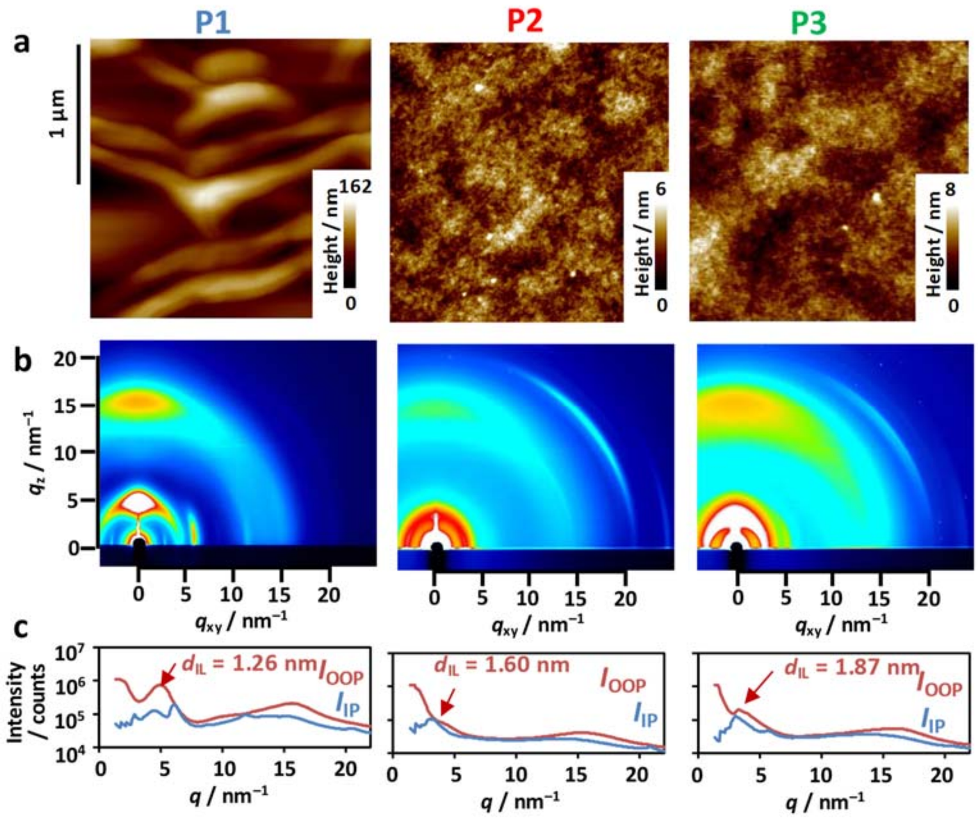

Fig. S5. (a) AFM height images of the pristine copolymer films. (b) 2D-GIXD images of the pristine copolymer films. (c) Out-of-plane (OOP, red line) and in-plane (IP, blue line) profiles. The interlamellar distances (d_{IL}) are appended with the arrows.

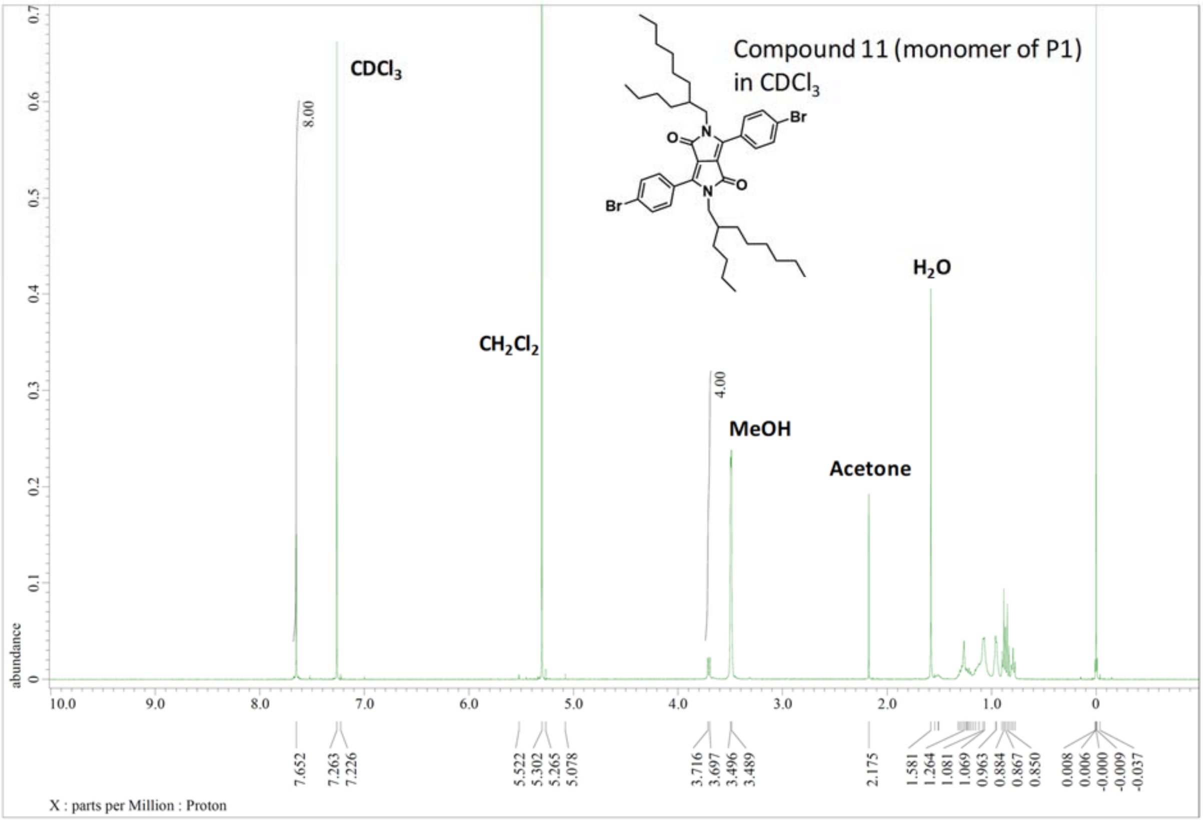

Fig. S6. ^{1}H NMR spectra of compound **11** (monomer of P1). CH_2Cl_2 and MeOH are included during crystallization. Acetone for washing may remain in a tube.

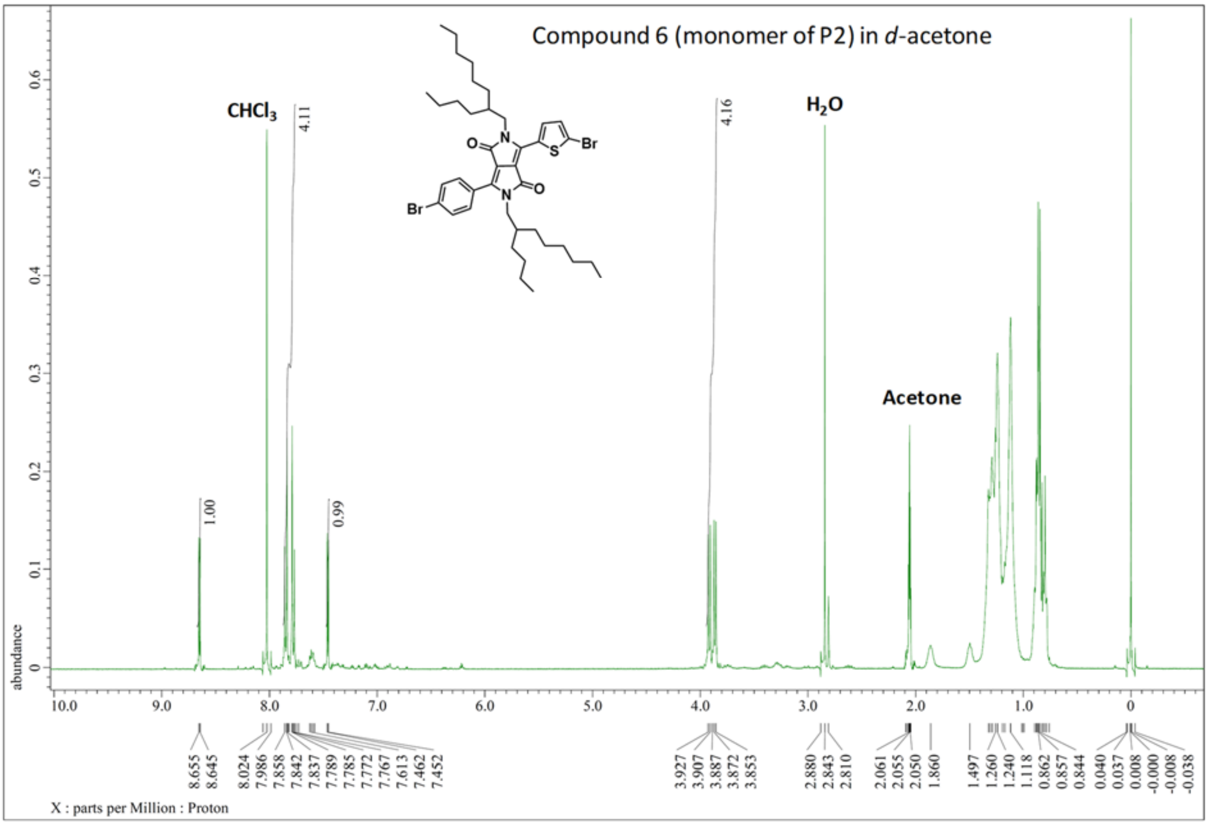

Fig. S7. ^{1}H NMR spectra of compound **6** (monomer of P2).

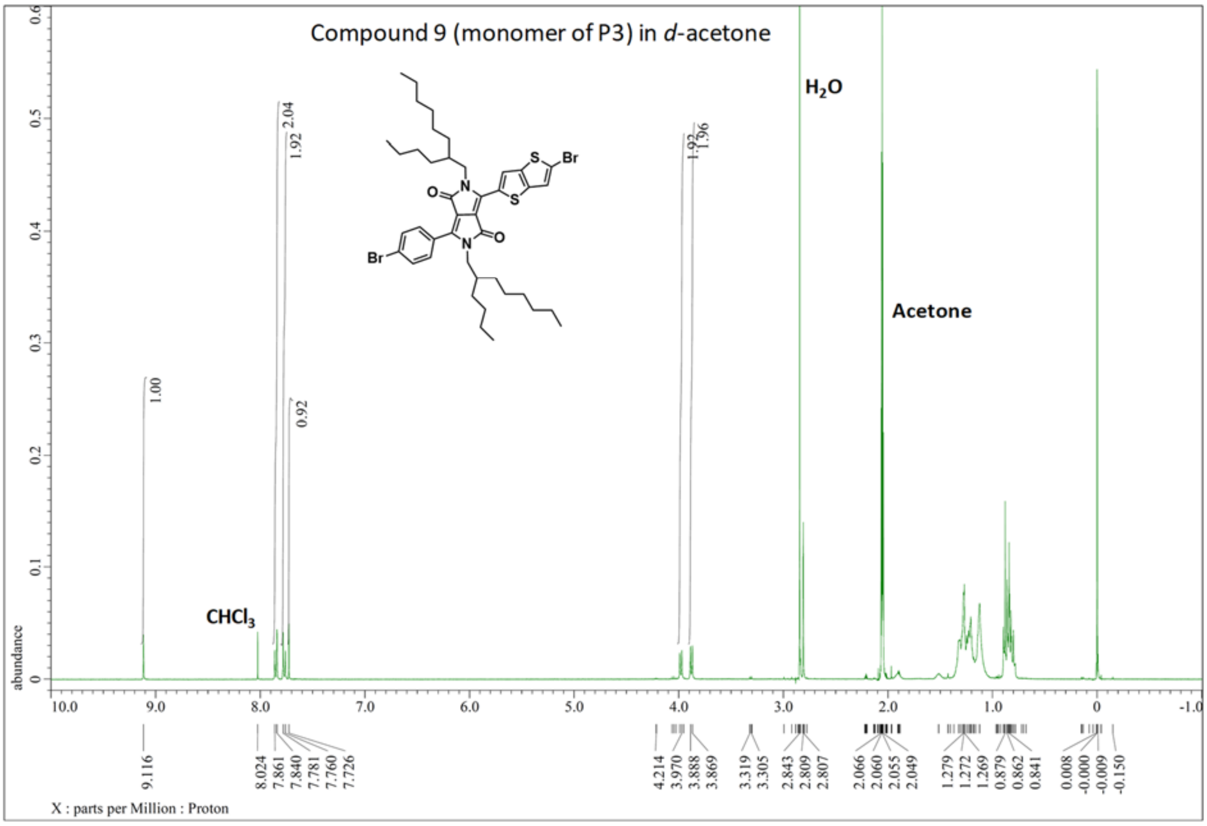

Fig. S8. ^{1}H NMR spectra of compound **9** (monomer of P3).

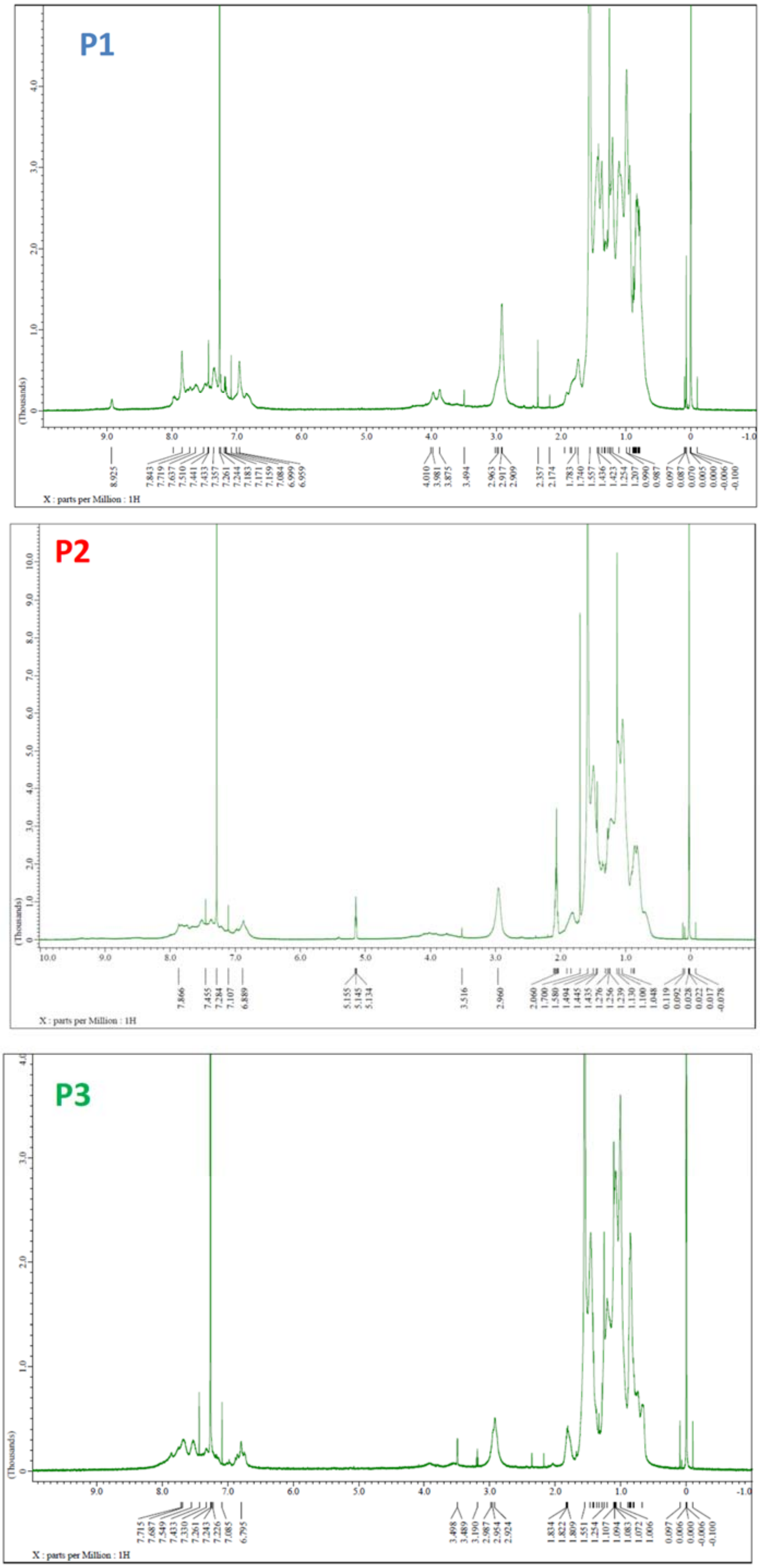

Fig. S9. ^{1}H NMR spectra of (a) P1, (b) P2, and (c) P3 in $CDCl_3$.
